# Studies of the Modular COsmic Ray Detector (MCORD) Using an Automatic Temperature Control Loop to Maintain Constant Gain Parameters of Semiconductor SiPM Photomultipliers

**DOI:** 10.3390/s26144356

**Published:** 2026-07-09

**Authors:** Marcin Bielewicz, Michał Kiecana, Aleksandr Bancer, Jarosław Grzyb, Martyna Grodzicka-Kobyłka, Tomasz Szczęśniak, Konrad Kopański, Wojciech Noga, Elżbieta Jaworska, Łukasz Kaźmierczak, Gabriela Saworska, Andrzej Brosławski, Piotr Mazerewicz

**Affiliations:** 1 National Centre for Nuclear Research, 05-400 Otwock, Poland; michal.kiecana@ncbj.gov.pl (M.K.); aleksandr.bancer@ncbj.gov.pl (A.B.); jaroslaw.grzyb@ncbj.gov.pl (J.G.); martyna.grodzicka@ncbj.gov.pl (M.G.-K.); tomasz.szczesniak@ncbj.gov.pl (T.S.); elzbieta.jaworska@ncbj.gov.pl (E.J.); lukasz.kazmierczak@ncbj.gov.pl (Ł.K.); gabriela.saworska@ncbj.gov.pl (G.S.); andrzej.broslawski@ncbj.gov.pl (A.B.); piotr.mazerewicz@ncbj.gov.pl (P.M.); 2 The Henryk Niewodniczański Institute of Nuclear Physics Polish Academy of Sciences, 31-342 Cracow, Poland; konrad.kopanski@ifj.edu.pl (K.K.); wojciech.noga@ifj.edu.pl (W.N.)

**Keywords:** SiPM, temperature loop, MCORD, Compton edge, temperature coefficient, cosmic ray

## Abstract

The Modular Cosmic Ray Detector (MCORD) is a modular scintillator-based system employing silicon photomultipliers (SiPMs) and FPGA-based digital signal processing, designed for applications such as cosmic muon detection, veto systems, and detector calibration support. In this work, we investigate the influence of ambient temperature variations on detector performance, with particular emphasis on SiPM gain stability. Several automatic temperature compensation loops were implemented to stabilize the operating voltage of the sensors. Based on controlled laboratory measurements, we evaluate the effectiveness of different control strategies, including variations in temperature averaging time and threshold response criteria. The performance of each approach is compared in terms of gain stability and response dynamics. We identify the optimal temperature control configuration for planned MCORD measurements and present recent modifications to the detector electronics, including updated software for Analog Front End (AFE) control. Additionally, we describe modifications made to the detector’s electronics since the previous publication, including new software developed to control AFE electronics.

## 1. Introduction

Work on the Modular Cosmic Ray Detector (MCORD) began around 2017 [1] as part of the development of a detection system for the Multi-Purpose Detector (MPD) in the Nuclotron-based Ion Collider fAcility (NICA) [2]. During the initial phase, the detector concept was developed, a demonstrator was constructed, and a Conceptual Design Report (CDR) and Technical Design Report (TDR) were prepared, forming the basis of this publication based on the Conceptual Design Report of the MCORD [3]. In subsequent years, the team from the National Centre for Nuclear Research (NCBJ) continued the detector development, performing detailed analyses and design optimizations. Currently, the detector is not dedicated to any specific experiment, making it a versatile instrument for a range of applications.

The MCORD detector is based on plastic scintillators produced by NUVIA [4], with 2 mm-diameter optical fibers from Kuraray [5], and light is read out from both ends of each tile using silicon photomultipliers (SiPMs) from Hamamatsu [6]. Each tile is equipped with its own AFE electronics, and each SiPM sensor includes a preamplifier and a temperature sensor. Signals from eight tiles are collected by a HUB module, which transmits them to FPGA-based digital electronics without modifying the analog signal. A detailed analysis and calibration of both the AFE and digital electronics has been described in a review publication on the MCORD detector [7], covering a full description of the MCORD detector with its block diagrams, ADC calibration methods, electronics stability, and the time required for the system to reach full stability after startup.

The current focus of the work is on the temperature dependence of MCORD operation. Due to the high sensitivity of SiPM gain to temperature variations [8,9,10,11], a Temperature Loop (TL) function was added to the AFE software. Under stable laboratory conditions, this effect is negligible; however, in variable environments, a supply voltage correction is required to compensate for temperature-induced gain changes. The gain stabilization of SiPM is generally achieved by using TL implemented in various ways (software or hardware); another method that can be used is to stabilize the ambient temperature around the SiPM or the entire electronics, but this method is much more difficult, more expensive, and requires the use of advanced cooling systems. In our case, we use software TL using data from temperature sensors. The TL measures the temperature near each SiPM sensor, averages the readings, and adjusts the sensor supply voltage based on a predetermined correction factor. The method for determining this correction factor is described in Section 3 (SiPM Coefficient Factor Measurement).

In this paper, we describe the development of the new AFE software (Section 6), with a particular emphasis on the operation of the TL (Section 7). Gain variations as a function of temperature and software parameters were evaluated using the Compton Edge (Section 4 and Section 7). All measurements were conducted in a climate chamber providing full control over ambient temperature (see Section 2). Finally, in Section 8, we present a comparison of system performance with and without the TL and assess the effect of different TL parameters on detector stability, identifying those crucial for its optimal operation.

## 2. Measurement System and Procedure

**Figure 1:**
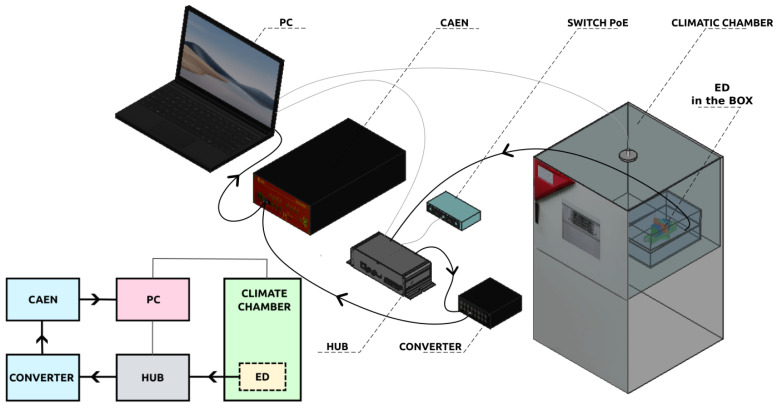
Visualization of the measuring system. The thick line shows how the measurement signal travels from the analogue front end (AFE) in the Equivalent Detector (ED) to the computer. The other lines show that some devices can be configured using a computer or via an Ethernet network.

The measurement setup used for the planned experiments is shown in Figure 1. Environmental tests with controlled temperature changes were carried out in a Binder MK 53 climate chamber [12] with internal dimensions of 40×40×33 $cm^{3}$ and a temperature range from −40 °C to 180 °C. Measurements to determine the proportionality coefficient (dV/dT) in the TL (Presented in Section 3) were performed in the temperature range between 10 °C and 35 °C, but measurements to check the operation of the TL were performed at temperatures between 15 °C and 30 °C (which is the typical operating range of the detector). The chamber allowed for the fully automated execution of pre-programmed measurement sequences, including controlled temperature ramps at specified rates. The computer was connected only to upload the measurement program and did not directly control or monitor the temperature during the experiments.

Due to the large size of a single MCORD detection slab [3], it was not possible to place it directly inside the climate chamber. To overcome this limitation, a special smaller-scale Equivalent Detector (ED) was prepared. Its construction is described below. The ED is housed in an aluminum enclosure, mimicking the original MCORD desk housing, and the entire assembly is placed inside the climate chamber. Measurement and control signals exit the chamber through a single cable connected to the MCORD HUB. The analog detector signals (measurement data) are transmitted without modification—signal amplification is performed inside the chamber by the AFE electronics—before reaching the ADC and analyzer (CAEN DT5730SB [13]). After each measurement, the collected data are transferred to a PC for offline processing and further analysis.

**Figure 2:**
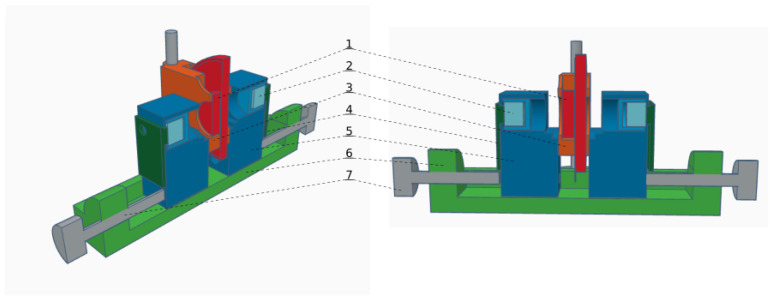
A three-dimensional ED model. The following symbols are used in the figure: SiPM sensors placed on electronic boards consisting of (4)—dark green planes, scintillators; (2)—light blue cylinders, Na-22 radiation sources; (1)—red discs, mounted, light green; (6) clamping screws; (7)—gray mountings for radiation sources; (3)—orange detector mounts; (5)—dark blue.

For measurements in the climate chamber, a special ED, consisting the radiation source holder (which can hold 1 to 3 sources) located exactly in the center between them, was developed (see 3D cross-sections in Figure 2). The ED was 3D-printed, allowing full adaptation to the dimensions of the mounted components. It accommodates two small electronic boards equipped with temperature sensors and SiPMs at opposite ends, small cylindrical scintillators made of the same material as used in the MCORD detector, and gamma radiation sources positioned along the central axis. For these tests, gamma radiation sources were used instead of cosmic rays due to the extended measurement times and the small scintillator dimensions. The ED design allowed for precise positioning of the radioactive sources along the measurement axis and, if required, adjustment of the distance between the scintillators using screws.

Two (in order to increase the count rate) Na-22 sources with an activity of 1 MBq (in 2018) were employed. The energy spectrum of this isotope contains two characteristic gamma lines: 1274.5 keV, originating from the de-excitation of the excited daughter nucleus Ne-22, and 511 keV, resulting from positron–electron annihilation following the $\beta^{+}$ decay of Na-22. Since the 511 keV quanta originate from annihilation process, they are emitted collinearly in opposite directions, making them particularly useful for coincidence measurements. The application of coincidence detection significantly suppressed the background contribution and enabled the registration of events only when simultaneous interactions were detected in both SiPM sensors (master and slave).

SiPM sensors are highly sensitive to changes in temperature, directly affecting the gain of the sensor signal. To filter out noise, we set the ADC lower threshold at channel 300 and upper threshold at channel 900 (1024 channels spectra). Consequently, the proposed detector must maintain a stable SiPM gain regardless of ambient temperature fluctuations. To evaluate both the gain variations of the SiPM photodetectors and the behavior of the scintillators under changing temperatures, the Compton edge in the energy spectrum recorded from the scintillators was used (see Section 4). Based on this approach, a dedicated TL was implemented in the detector control software. The TL adjusts the SiPM bias voltage to ensure that the position of the Compton edge remains stable, independent of temperature changes. The TL software continuously measures the temperature near each SiPM photodetector, averages the readings, and, when the temperature variations exceeds a predefined threshold, corrects the sensor’s bias voltage by the required amount.

## 3. Temperature Coefficient Factor Measurement

One of the key parameters of silicon photomultipliers (SiPMs) is their temperature coefficient, which defines the change in breakdown voltage as a function of temperature [8,9,11]. Even small temperature variations can lead to noticeable changes in the breakdown voltage; consequently, if the bias voltage remains constant, this can lead to variations in the SiPM gain. In the MCORD detector, Hamamatsu S13360-3075PE photodetectors (3 mm × 3 mm, pixel size 75 μm) [6] were used. According to the manufacturer, the nominal temperature coefficient is 52 mV/°C. To verify this value for our sensors, a series of measurements was carried out for two samples from the delivered batch (SN4680 and SN4683). The current–voltage (I–V) characteristic were measured using a Keysight B2900A source-measure unit [14], with the sensors placed inside a Binder MK 53 climatic chamber. The chamber temperature was controlled with an uncertainty of ±0.2 °C. For sample SN4680, the measurements were performed in the temperature range from 0 °C to 30 °C in 2.5 °C steps, while for sample SN4683, the range was from 0 °C to 25 °C in 5 °C steps.

**Figure 3:**
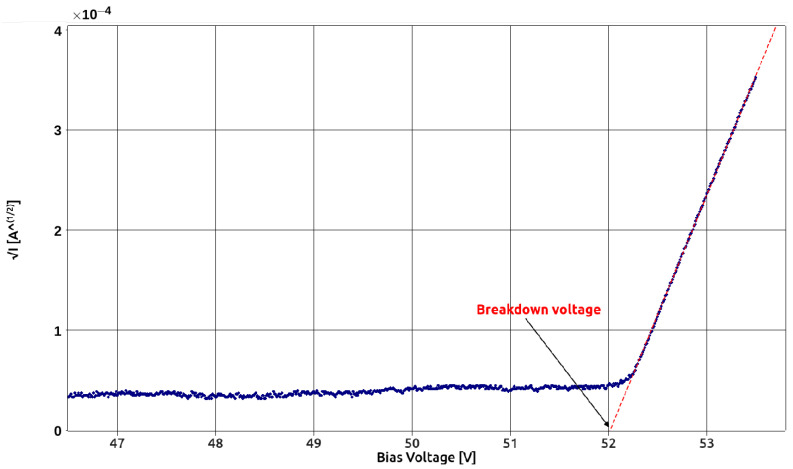
Current–voltage (I–V) characteristic.

**Figure 4:**
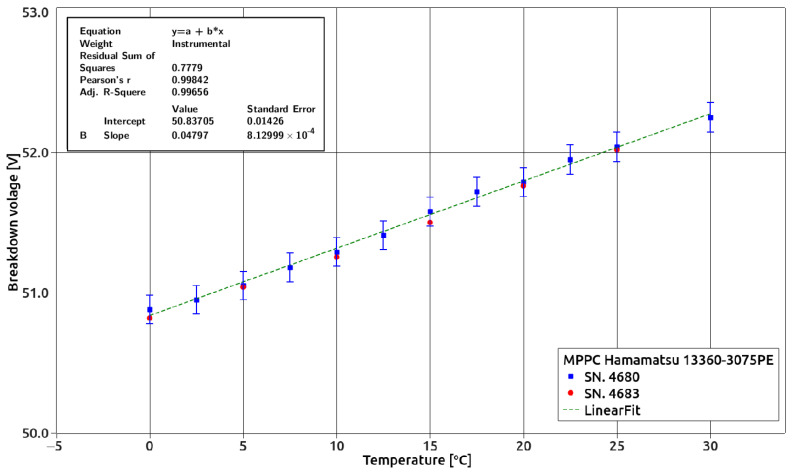
Breakdown voltage vs. temperature.

The obtained I–V characteristic is presented in Figure 3. In the figure, the x-axis corresponds to the voltage applied to the SiPM, while the y-axis represents the square root of the dark current measured at a given bias voltage. As shown, the current increases sharply once the bias voltage exceeds a certain value, corresponding to the beginning of the full avalanche multiplication (Geiger mode). The data points above this voltage (marked in blue) can be well fitted with a straight line. The breakdown voltage $V_{br}$ was defined as the intersection point of this line (red line) with the voltage axis. The dependence of the breakdown voltage on temperature is shown in Figure 4. From a linear fit to the data, the measured temperature coefficient was determined to be 48 mV/°C. The errors of the results presented here were determined by calculating the standard deviation of the series of measurements performed in the same conditions. As can be seen from the laboratory measurements, the supply voltage correction factor we obtained is only slightly lower than the manufacturer’s specification (52 mV). Furthermore, since correcting the supply voltage by 1 mV to 2 mV is impractical, based on these two values, we can assume that our factor is approximately 50 mV per step. The above measurements and analyses focused solely on the SiPM sensor itself. In reality, it is part of a measurement system consisting of the AFE electronics and a scintillator. For this reason, a series of additional measurements were performed to test the behavior of the entire system, including the SiPM sensor, in the same climatic chamber to receive MCORD full device temperature sensitivity.

The sensitivity of the MCORD system changes with temperature variations, not only due to the SiPM sensor itself. In real-world conditions, the presence of the plastic scintillator and the entire AFE electronics must also be taken into account. This set operates as a complete measurement system in close proximity to the SiPM sensor and is affected by the same temperature changes. Testing entire MCORD detector boards under controlled temperature conditions was not possible due to the size limitations of the climatic chamber used. The new much smaller ED set was placed in an aluminum box (replacing the MCORD detector board housing) during chamber measurements.

Measurements of breakdown voltage as a function of temperature were used to determine the correction factor for the SiPM sensor itself. We believe that for the entire system, a good method for determining the correction factor is to measure the change in the position of the Compton edge as a function of temperature in the spectrum of radioactive Na-22 (511 keV annihilation quanta from $\beta^{+}$ decay). Due to the low event statistics (two Na-22 sources but with low activity), a novel method for determining the Compton edge value and estimating measurement error with low measurement statistics was developed and used (see Section 4, Compton Edge). The ED model of the MCORD detector was tested over a temperature range of 15 °C to 35 °C. Within this range, the value of the SiPM supply voltage change was tested to maintain the reference Compton edge level for a system composed of two sensors (master and slave—the two ends of the MCORD detection board).

**Figure 5:**
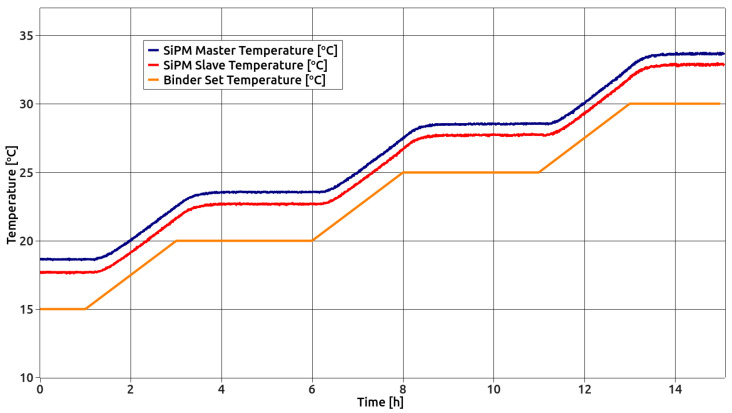
The graph compares the temperature set in the climate chamber and the temperature read by the SiPM sensors.

The measurement procedure was as follows. Measurements were taken at the climatic chamber temperature points of 10, 15, 20, 25, 30, and 35 °C. The system is assumed to operate at room temperature, defined as a climate chamber setpoint of 20 °C. Due to the heat generated by the nearby AFE electronics, the temperature measured directly on the SiPM sensor board is approximately 23 °C. This temperature is taken as the reference operating point in the subsequent analysis. The differences between these temperatures are due to the fact that the AFE electronics generate heat during operation; in addition, the system is housed in an aluminum enclosure. This difference, once the temperature has been changed and stabilized, remains approximately constant across the entire temperature range, measuring about 3.5 °C for the SiPM Master and 2.5 °C for the SiPM Slave (Figure 5). The graph shows that after a change in the chamber temperature, it took approximately 0.5 h for the temperature inside the ED enclosure to stabilize. A similar phenomenon occurs in the actual MCORD detector board. In the remainder of this article, we will primarily use the temperature value reported by the AFE sensors.

**Figure 6:**
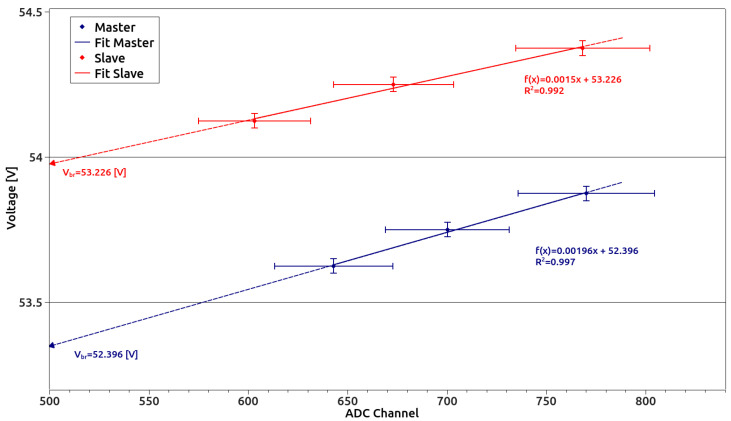
The dependence of the required bias voltage for the Compton edge position shift, recorded for SiPM Master (blue) and Slave (red) at 25 °C.

As the temperature in the climate chamber increases at a constant SiPM bias voltage, the Compton edge value decreases (the energy spectrum shifts to the left, towards the lower channel number). In order to control the gain of the detector, we arbitrary selected the reference point to be the Compton Edge position (channel number) at 20 °C at the voltage of 53.5 V for the SiPM Master and 54 V for the SiPM Slave (these values will be referred to as reference values later in the article). To find the bias voltage change required to maintain the Compton edge position at the temperatures other than 20 °C, several measurement points were recorded around the reference value for a given temperature. Examples of these measurements for SiPM Master and Slave are presented in Figure 6, showing the relationship between the voltage applied at 25 °C and the Compton edge position (expressed in ADC channels). Voltage uncertainty was determined by comparing the AFE circuit with a Keithley 6517A precision electrometer/voltmeter [7] and was found to be 0.025 V over the entire range of measured voltages. The uncertainty with which the Compton edge position was determined is described in detail in Section 4. In general, the unchanged peak position is equivalent to the unchanged SiPM gain, which in turn indicates operation at a constant overvoltage. Maintaining a constant overvoltage requires accurate knowledge of the detector’s breakdown-voltage dependence on the temperature. It is worth emphasizing that, in our case, the breakdown voltage refers to the entire detector system rather than to the SiPM alone. Therefore, it also incorporates effects associated with the scintillator properties and the readout electronics.

The detector’s breakdown voltage was determined by fitting a linear function y = ax + b to the data shown in Figure 6. At the breakdown voltage, the detector gain is equal to zero (i.e., the Compton edge position corresponds to channel 0); therefore, the parameter b is the quantity of interest.

**Figure 7:**
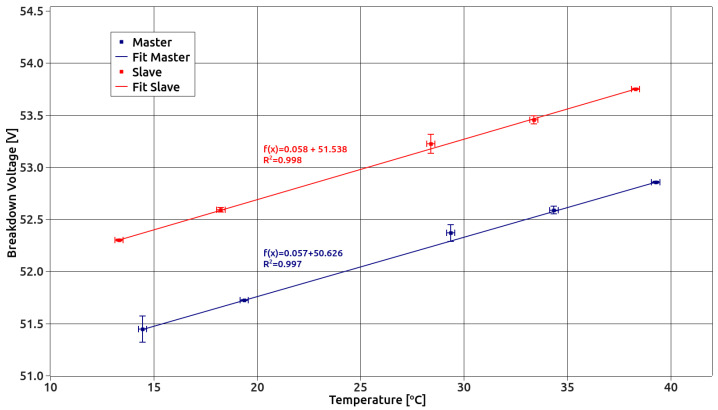
The dependence of the full detector breakdown voltage on the temperature. Linear fitting allows for the determination of the temperature coefficient.

The above-described series of measurements and detector’s breakdown voltage calculations was performed for six consecutive temperature changes, and the resulting voltage values were plotted on a single graph (Figure 7). In this graph, the slope of the linear function, adjusted for the six measurement points, is the desired coefficient factor for the entire system. Measurements were collected separately for the AFE master and slave electronics, but ultimately, a single average value was adopted. The measurement results shown in Figure 7 show that the coefficient value for the entire system (SiPM, scintillator, AFE electronics, housing) is significantly higher than the value determined for the SiPM alone and is approximately 58 mV/°C. This coefficient value was used in all further measurements. The measurement uncertainties associated with the temperature measurement are 0.2 °C. The detector’s breakdown voltage uncertainties were determined from the uncertainty of the linear fit obtained using the least-squares method. The measurement uncertainty of the temperature coefficient was calculated using a linear regression procedure and has been estimated at 1 mV/°C.

## 4. Compton Edge and Error Analysis

To determine the Compton edge by comparing measurement results with various analytical functions, we assumed that the following function (see Equation (1)) fits the measurement results well enough to determine the parameters for the voltage correction procedure for the SiPM sensors in our detector:

(1) $$I\left(E\right)=\left\{\begin{array}{ccc} \frac{a-c}{b^{2}}E^{2}-2\frac{a-c}{b}E+a & \; & \mathrm{if}\;E\leq b\\ c & \; & \mathrm{if}\;b<E\leq d\\ -\frac{c}{e-d}E+\frac{ce}{e-d} & \; & \mathrm{if}\;d<E\leq e\\ 0 & \; & \mathrm{if}\;E>e \end{array}\right.$$

where the function parameters were chosen so that on the boundaries of the first (E≤b) and second (b<E≤d) intervals, both the function values I(E) and their derivatives $I^{\prime }\left(E\right)$ were equal. For the remaining interval boundaries, we assumed that the condition that the function values were equal on the boundaries of the intervals was met. Next, we assumed that the function interval in which it takes on a constant value *c* corresponds to a local maximum, and the energy value for the Compton edge lies on the line describing the fitted function for the interval (d<E≤e) and corresponds to a radiation intensity of 75% of the value of the radiation intensity’s local maximum. For the third interval, a fit to a linear function was adopted, which significantly simplifies the calculations, and the use of an exponential function in this case gives the same result in practice.

**Figure 8:**
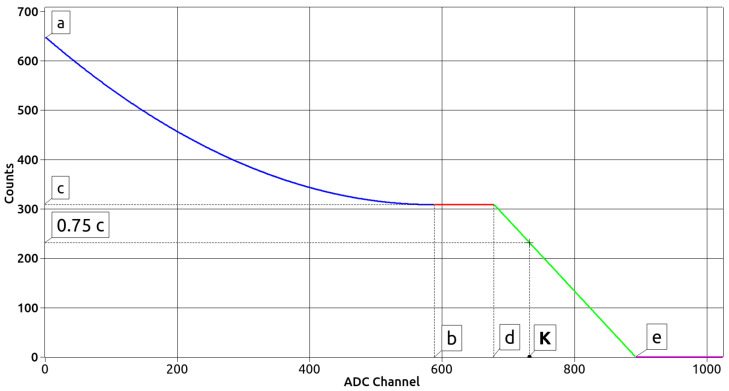
A function defined by Equation (1) was fitted to the measured data. It consists of 4 parts: the first (dark blue line) interval (0,b] is described by a quadratic function that starts at the point (0,a) and ends at the point (b,c); at interval (b,d] there is a plateau with value *c* (red line), which on the interval (d,e] turns into a decreasing linear function (green line). For values greater than *e*, the number of detected gamma quanta decreases to zero (magenta line). We assume that the Compton edge energy corresponds to 75% of the plateau with value *c*.

**Figure 9:**
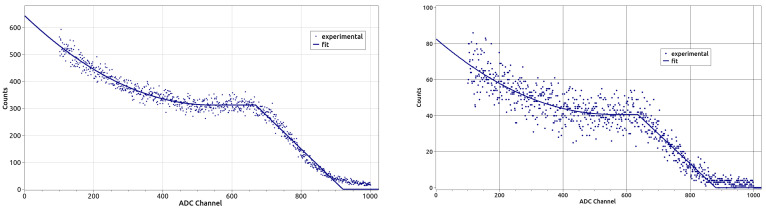
Comparison of measurement data (dots) with the relationship fitted to them (line). The graph shows the typical shape of the measured Na-22 coincidence spectrum (511 keV line) for our detector and the function fitted to it, defined by the formula Equation (1) in case of very long (2 days) measurement (**left**) and in the case of short (8 h) measurement (**right**) (an example of the low statistics spectrum and the Compton edge fitting performed in the case).

The parameters of the fitted function are shown in the graph (see Figure 8). The next figure (Figure 9) shows an example of such a fit to real data. These assumptions mean that the energy of the Compton edge is given by the following formula:

(2) $$E_{com}=\frac{3}{4}d+\frac{1}{4}e$$

The procedure for determining the fitting parameters is implemented using the NonlinearModelFit function in Wolfram Mathematica [15]. It can also be used to obtain the measurement uncertainties of the individual fitting parameters. Knowing these uncertainties, the uncertainty of determining the Compton edge can be calculated using the total differential method. Applying this method leads to the following formula:

(3) $$\Delta E_{com}=\frac{3}{4}\Delta d+\frac{1}{4}\Delta e$$

where Δd and Δe are the uncertainties obtained using the previously mentioned Mathematica function. In the rest of this article, this uncertainty $\Delta E_{com}$ will be referred to as statistical uncertainty. In addition to the aforementioned statistical uncertainties resulting from the Compton edge determination method, there are also uncertainties related to the limited precision of the voltage setting on the SiPM and the limited accuracy of the temperature reading. The errors in determining the temperature and voltage are small compared to the other errors and they are included in the complete error described at the end of this section.

### 4.1. Dependence of the Statistical Error Limit on the Measurement Time

**Figure 10:**
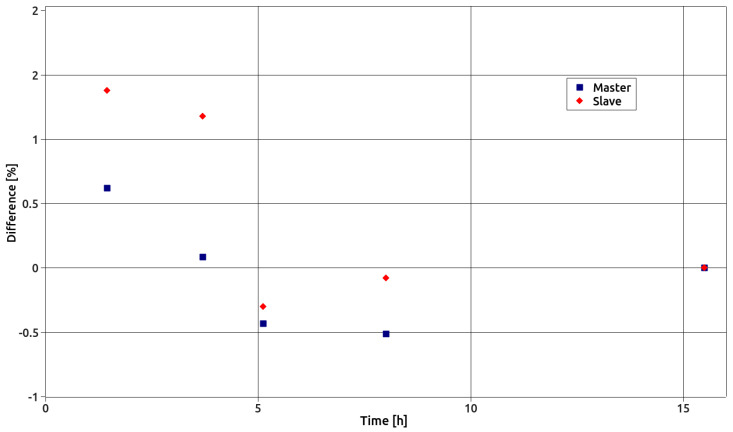
Measurement at a constant temperature of 25 °C. The graph shows how the Compton edge value converges over time to a value determined after approximately 16 h (the y-axis shows the percentage difference between the value measured after 16 h and the values measured in earlier time intervals).

The measurements were made using two Na-22 sources with low activity. To determine the recommended minimum measurement time, a series of identical measurements were performed, varying only the data acquisition duration. Figure 10 shows the results of these measurements after 1, 4, 5, 8, and 16 h, with the 16 h measurement taken as the reference level. We see that after 6–8 h, the range of spectrum variations drops to less than 0.5% compared to much longer measurements.

In subsequent measurements, we assumed this as the minimum measurement time. After 8 h of measurement, the number of counts near the upper plateau of the fit was only about 30–40. Our fitting method performed very well even for such low measurement statistics, as seen in Figure 9, right side. However, to increase measurement confidence, we ensure that most measurements have significantly better statistics than 100 on the upper plateau (see Figure 9, left side).

### 4.2. Repeatability of Measurements

**Figure 11:**
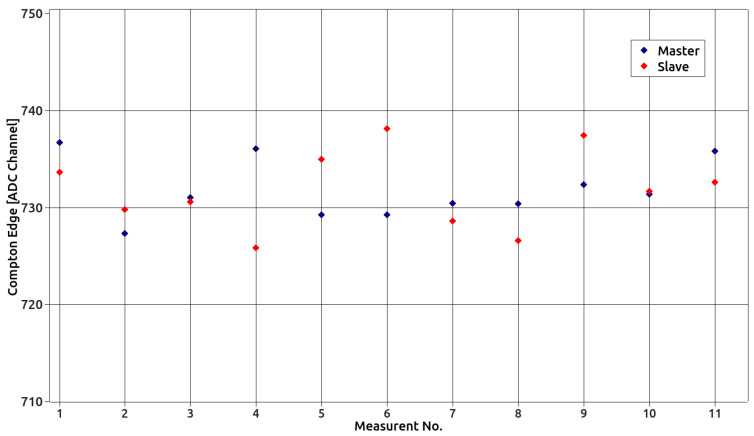
Measurement of the Compton edge at a constant temperature of 30 °C in a climate chamber. The same six-hour measurement was repeated 11 times. It can be seen that the differences between the measurements are small and that there is a high degree of repeatability between measurements.

To verify the repeatability of the results in our measurement setup, 11 identical measurements were performed. Each measurement lasted 6 h (corresponding to the collected statistics at the upper plateau of greater than 30 counts) and was performed at a constant temperature of 30 °C. As can be seen in Figure 11, the dispersion of the measurement points is relatively small, resulting in an error of approximately 0.5%. For AFE Master electronics, the dispersion was 0.42%, and for Slave electronics, 0.55%. We can see that the repeatability of the results is good even with low statistics.

### 4.3. Measurement Uncertainty Arising from Differences Between Repeated Measurements Carried out Under Similar Conditions

We estimate this uncertainty based on the average differences for various series of measurements. Some of the measurements, which initially lasted several hours, were repeated with a measurement time of 72 h to obtain improved statistical results. These measurements were taken after removing the source from the climate chamber and ED, and then by repositioning it in the ED and placing it back in the climate chamber. We believe that if we take the average of the differences between the Compton edges determined in this way, it will be a good estimate of the impact that minor imperfections in sample positioning and other factors may have. The uncertainty of Compton Edge ΔE value determined in this way is approximately 12 ADC channels (1.91% to 2.29%).

### 4.4. Measurement Uncertainty Based on Manual Parameter Adjustment

**Figure 12:**
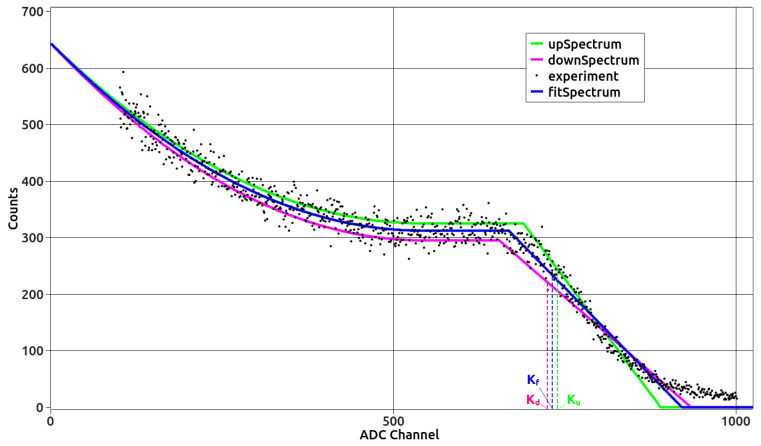
Three types of numerically fitted curves to measurement data (using Mathematica). The difference between $K_{u}$ and $K_{d}$ is the measurement uncertainty for “Manual Parameter Adjustment” estimated by us.

Another contribution to estimate measurement uncertainties is to manually determine the extreme parameters for one of the measured curves, for which the fit to the measurement data seems reasonable. Figure 12 shows a comparison of the curve described by Equation (1), numerically fitted to the experimental data (see Section 4), and two functions manually fitted in such a way that they take extreme but reasonable-looking values below and above the numerically fitted function. Based on these parameters, two Compton edge values ($K_{u}$ and $K_{d}$—upSpectrum and downSpectrum on Figure 12) were calculated, and the difference between them was used as the basis for calculating the relative uncertainty. This value, expressed as a percentage (2.27%), forms the basis for calculating the measurement uncertainty for all measured relationships.

### 4.5. Summary of Measurement Errors

We assumed that the uncertainty with which we measure the Compton edge is the sum of the difference between measurement results repeated under very similar conditions and the statistical uncertainty arising from the fact that the fitted function parameters (Equation (1)) are subject to uncertainties, as well as a manually determined uncertainty based on an assessment of how reasonable the fit appears for the various parameters.

## 5. MCORD Electronic Correction

**Figure 13:**
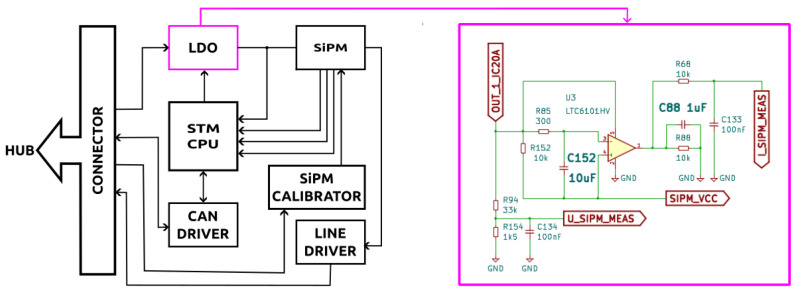
Block diagram of the AFE circuit with modifications marked (bold text) in the simplified electronic diagram of the LDO block.

**Figure 14:**
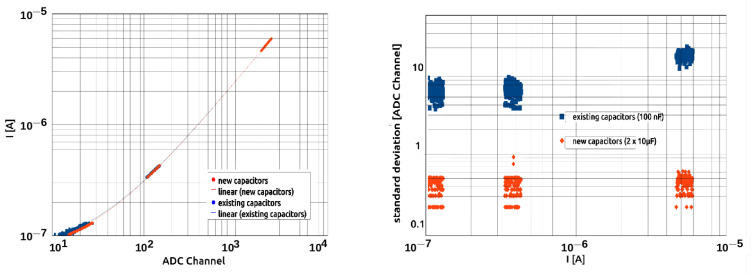
(**Left**): The relationship, measured for the purpose of calibrating the AFE, between the current measured by the AFE (the average of several measurements in the ADC channels) and the current calculated using voltage measurements on three reference resistors—a detailed description can be found in the article [7]. (**Right**): The standard deviation from the mean current value for measurements at various current levels.

As shown in article [7], the AFE electronics we used so far and the converters ADC within it generated a certain level of noise (up to 20–30 bits), which prevented the precise determination of the supply voltage. We decided to modify the existing electronic circuit to mitigate this effect. The changes described in this section are supplementary to the information provided in article [7], and we will not refer to this topic in the rest of this article. During calibration measurements of AFE circuits for very low SiPM current levels (on the order of $1.0\times 10^{-7}$ A, corresponding to values of several tens of bits on the ADC), significant signal fluctuations were observed, characterized by a standard deviation at the level of several bits on the ADC. This resulted in poor repeatability of results at very low currents. After analyzing the AFE electronic circuit, we concluded that the cause was too short averaging time of the acquired signal at the U3 operational amplifier (Figure 13), which is part of the LDO block (Low DropOut regulator—built-in linear voltage regulator with low voltage drop). To improve measurement accuracy, capacitors C88 (1 μF) and C152 (10 μF) were added to the LTC6101HV operational amplifier circuit. This modification resulted in an increased measurement time constant and a substantial reduction of current fluctuations. The standard deviation decreased to below 1 bit. For low current levels, the standard deviation was comparable to the measured signal value, whereas after adding the capacitors, it decreased to a few percent of the measured value. In Figure 14, on the left, we see that the mid- and high-range current measurements were equally accurate before and after the changes (the red and blue points overlap and form lines). However, for low currents, the spread of the blue points (the system before the change) is much greater than that of the orange points (the measurement after the change). Low current intensities are also particularly important when determining the breakdown voltage of SiPMs. The introduction of the capacitors significantly enhanced the repeatability and linearization of calibration measurements, especially for low current intensities (Figure 14, right side).

As a result of using an additional capacitor in our circuit, the noise level decreased by a factor of 10. Current measurements performed using an internal meter are much more accurate. Most importantly, the measurement values for small currents are repeatable.

## 6. Update of AFE and HUB Software

To date, measurements using the MCORD detector have utilized electronics software written by individuals from the Warsaw University of Technology during the project’s initial stages (see article [7]). After consultations, it was decided to not only modify the TL portion of the software but also rewrite the entire software, including the detector activation section.

### 6.1. HUB Firmware and Control

The firmware is written in micropython [16]. Each task is run by the UASYNCIO library to achieve multitasking. HUB has an SD card, where loaded firmware and data from AFEs can be stored. HUB can be controlled via Ethernet, thanks to the server task. If AFE is found, then configuration is read from the configuration files. HUB has turned on Independent WatchDoG (IWDG).

The HUB Main Loop is dedicated to manage AFEs. It is divided by subtasks:

- Dequeue CAN message—it gathers the last message (if available) from the CAN Handler task.
- Discover devices—it aims to find all connected AFEs.
- Process received message—it processes received messages, obtained by the first subtask.
- Managing AFE—it manages each discovered AFE.

The HUB server manages communication over Ethernet. The request, for early development, is in JSON format. It should contain the name of the procedure (e.g., close all, get all configuration, power off, set parameter, set voltage) and its values. After receiving such a request, the client should wait for the response. Sometimes it can take a long time (a few seconds) due to other tasks as well as on-HUB and on-AFE tasks. The periodic task contains functions to manage logger (saving data on a SD Card) and to print by UART (USB).

### 6.2. AFE Firmware Overview

The AFE Firmware is written in C with the STM32F0 HAL (Hardware Abstraction Layer) library [17]. It operates as a state machine that continuously monitors for incoming CAN messages and measurements. All interactions with the AFE module are performed via the CAN bus. The device uses standard CAN 2.0A frames with 11-bit identifiers. For each of the 8 ADC channels, the user can independently configure data processing parameters. Upon receiving a valid command, it performs the requested action—such as reading last measurement, setting DAC, or updating configuration parameters. The firmware also manages several autonomous tasks, including the following:

- Periodically acquiring data from all configured ADC channels and storing it in circular buffers.
- Executing the temperature compensation feedback loop (Temperature Loop).
- Managing the gradual ramping of DAC Voltages.
- Transmitting periodic status and data messages if configured to do so.
- Listening for incoming CAN messages and executing commands.
- Managing software watchdogs.

The ADC can be triggered either by a configurable TIM (hardware timer) with DMA (Direct Memory Access) or by software. The previous version was triggered by software (polling), and calibration was performed for that option. After a software update, calibration differed for the ADC when using DMA, so the ADC is now triggered by software (polling). The new polling method allows other tasks to continue during polling, but it can be enabled if communication is more critical than measurements. During each ADC polling cycle, every ADC channel is measured. However, the timestamp and raw measurement value are stored in the circular buffer only at the programmed intervals.

### 6.3. Temperature Loop Software

A critical feature for SiPM operation is the automated temperature compensation loop. The gain of a SiPM is linearly dependent on the over-voltage, which in turn depends on the breakdown voltage. Since the breakdown voltage varies with temperature, the bias voltage must be adjusted to maintain a constant gain. The AFE module implements a closed-loop feedback system to perform this compensation automatically. The user configures the loop with the following parameters, independently for each channel:

- Temperature Coefficient (dV/dT): The change in bias voltage required per degree Celsius change in temperature (V/°C).
- Optimal Operating Point ($T_{opt}$, $V_{opt}$): The desired reference temperature and the corresponding optimal bias voltage.
- Dead-band (ΔT): A temperature window around the last set point within which no voltage adjustment is made, preventing oscillations.

The firmware periodically reads the temperature from a designated sensor channel; compares it to the last recorded temperature; and, if the change exceeds ΔT, it calculates the new required bias voltage using the formula:

(4) $$V\left(T\right)=V_{opt}+\frac{dV}{dT}\left(T-T_{opt}\right)+V_{corr},$$

where *T* is the averaged temperature and $V_{cor}$ (manually added offset voltage value—default value is zero). The DAC output is then updated to this new value. The entire loop can be enabled or disabled. To protect the connected detector from sudden voltage changes, the firmware includes a DAC ramp controller. Instead of setting the DAC output directly, the user can set a target voltage. The firmware will then automatically ramp the voltage from its current value to the target value in small, configurable steps. The ramp rate (e.g., step size and time between steps) can be configured, ensuring a smooth and safe transition. This feature is active for both manual voltage changes and adjustments made by the automated temperature compensation loop. The default value of the ramp is set to 100 bits per 100 ms, but it can be changed by CAN commands. For monitoring purposes, any ADC channel can be configured to periodically transmit its latest or averaged reading over the CAN bus without requiring a request from the host. The user can set the reporting period for each channel individually. This reduces the CAN bus load in systems where continuous polling is otherwise necessary.

## 7. Temperature Loop

**Figure 15:**
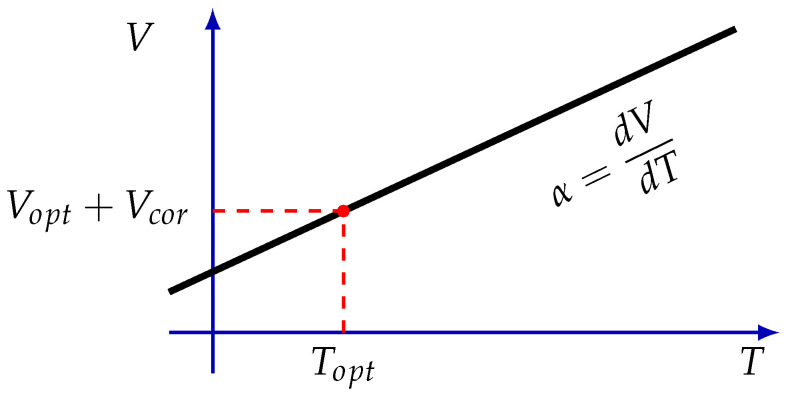
A straight line showing the relationship between the voltage across the SiPMs and temperature, parameterized by the coefficients used in the temperature control loop.

While establishing the operating assumptions for the entire AFE software, a definition of the operation of the TL was also prepared, i.e., how the supply voltage of the SiPM sensors will change with the temperature change. We want the voltage setting on the SiPM sensors to be corrected so that the data are on a straight line (Figure 15).

(5) $$V\left(T\right)=\alpha T+b,\alpha =\frac{dV}{dT},$$

where α is the slope of the line. This coefficient is known as the temperature coefficient. Its value can be taken from the manufacturer’s technical documentation or determined by independent laboratory measurements (see Section 3). The *b* coefficient is calculated based on the condition that the line must pass through the optimal point:

(6) $$P(T_{opt},V_{opt}+V_{cor}),$$

which leads to Equation (4), where dV is the voltage value by which the change should occur after a one-degree temperature change. *T* is the average ambient temperature of a given SiPM sensor and is determined using measurements taken by temperature sensors located near each SiPM sensor. $V_{opt}$ is the operating voltage determined at temperature $T_{opt}$. The $V_{opt}$ is called the optimal voltage, and it was described in [7]. Currently, the important information for us is that the value of this voltage at a given temperature is given, and our program retrieves it from the database as the reference voltage. It was assumed that the reference/optimal voltage was determined for a temperature of 25 °C. If these values for a given SiPM sensor are missing from the database, the program retrieves the average value for the remaining measured values.

**Figure 16:**
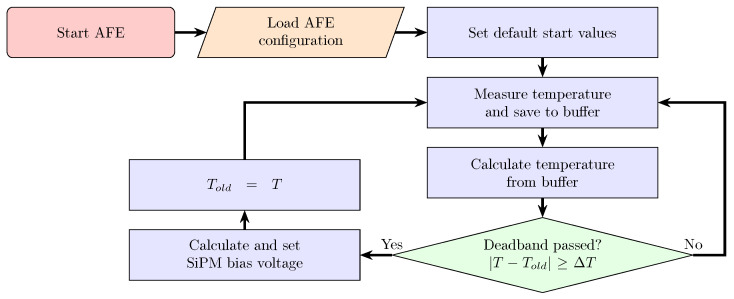
AFE Temperature Loop workflow.

$V_{cor}$ is the voltage (additional factor) by which we can correct the determined operating voltages if this is necessary for a given measurement (e.g., when the signal is saturated), or in case a systematic error not previously considered is detected. There are various ways to calculate the average temperature value. However, our procedure will also consider other parameters related to the average calculation method, such as the number of measurement points from which the average is to be calculated, the time over which these points will be collected, and the temperature change threshold (ΔT) that must be exceeded before the program (Temperature Loop—TL) performs a voltage correction. A simplified workflow diagram of the AFE TL is shown in Figure 16.

**Figure 17:**
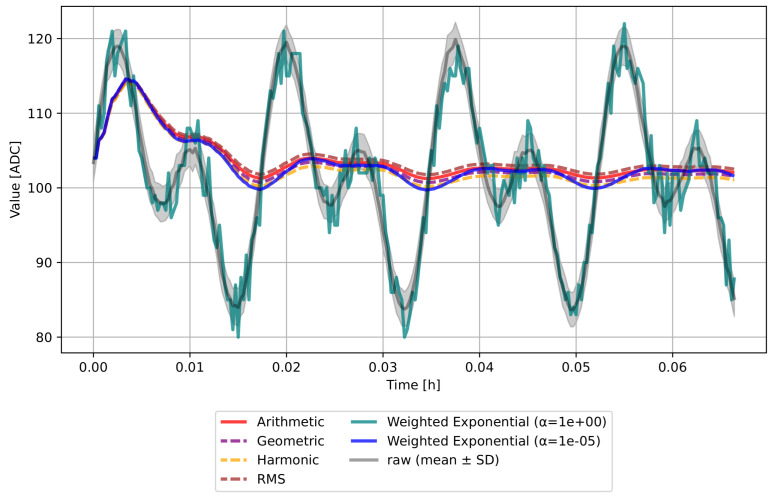
Comparison of temperature averaging algorithms. Settings: Buffer size: 256, save measur. every 1000 ms, sampling every 10 ms.

For the TL, a few averaging methods were prepared: arithmetic mean, weighted exponential moving average, geometric mean, harmonic mean, and root mean square. The libraries used in AFE were used to perform the simulation. The simulator incremented the time and used the given function to determine the ADC value:

(7) $$10(\sin\frac{2t}{\tau }+\sin\frac{t}{\tau }+2*0.2\left(2U\left(0,1\right)-\frac{1}{2}\right))+100,$$

for τ=10,000 and given time *t* in ms. Every step was recorded to the file as an “raw” (ADC sampling) and internally processed data by selected setting for the same time and ADC value. The simulator was intentionally prepared as a technical tool to test the algorithms used in the AFE. Figure 17 presents the algorithms used in simulation can be divided by 3 basic groups: arithmetic mean, weighted exponential, and low and high alpha. Using the weighted exponential, alpha can modify the importance of the latest measurements; in other smoothing methods, it mainly depends on buffer size and the time between measurements.

## 8. Measurement Results and TL Analysis

Below, we present the most important measurement results collected during a measurement session lasting several months. This duration was this long primarily due to the fact that we attempted to perform most measurements with high accuracy (see Section 4), which translated into long three-day measurements. By performing subsequent measurements, we aimed to verify the performance of the TL even with very long measurements. However, above all, we aimed to assess how various parameters of the loop itself affect its effectiveness. The new software for our electronics (see Section 6 and Section 7) allowed us to adjust a number of variable parameters affecting the performance of the TL. We wanted to demonstrate which of these parameters have a significant impact and which are less important. The parameters we adjusted included the method for calculating the average temperature, the duration of temperature measurements used to calculate the average, and the activation threshold (the minimum temperature change after which the loop corrected the supply voltage of the SiPM sensor). Our software also allows for an adjustment of the number of temperature measurement samples at a given time, but we did not test this variable in these measurements.

**Figure 18:**
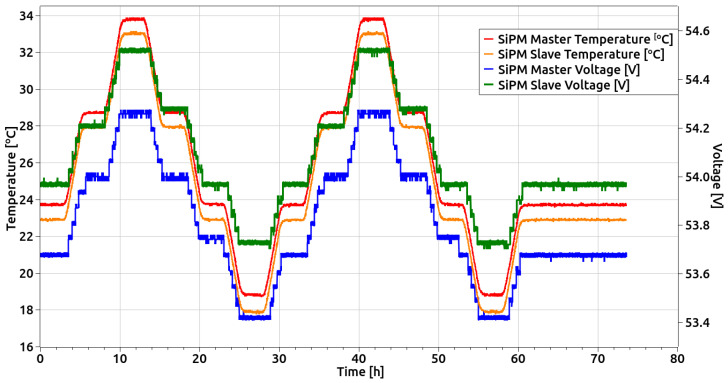
Example of the operation of the TL during multiple temperature changes and stabilization (SiPM sensor supply voltage compensation changes in ambient temperature), during an Example 3-day measurement in a climatic chamber.

**Figure 19:**
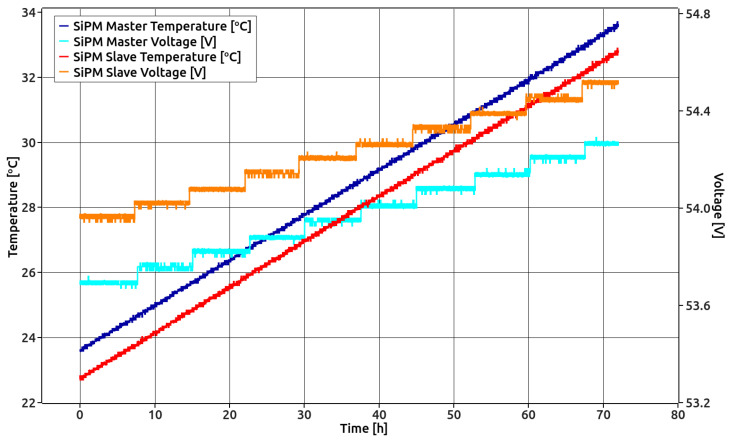
Example of TL operation during our real measurements (temperature change occurred only in one direction), during a 3-day measurement in a climatic chamber.

After completing preliminary measurements and analyses (see Section 3 and Section 4), the operation of the TL was first verified over a multi-day measurement cycle, during which the ambient temperature (inside the climatic chamber) changed repeatedly between 15 and 30 °C. Each 5 °C temperature change was followed by a period of several hours of constant temperature. Figure 18 shows the time-correlated temperature changes and changes in the SiPM supply voltage. It is also clear that voltage changes occurred only when the cumulative temperature change exceeded a preset threshold (in this case, 0.5 °C). This measurement demonstrated that the operation of the TL was always correct and stable over time. In subsequent measurements, the temperature change cycle shown in Figure 18 was not used; instead, a simpler cycle of only increasing temperature within the 20 °C to 30 °C range was used (Figure 19).

**Figure 20:**
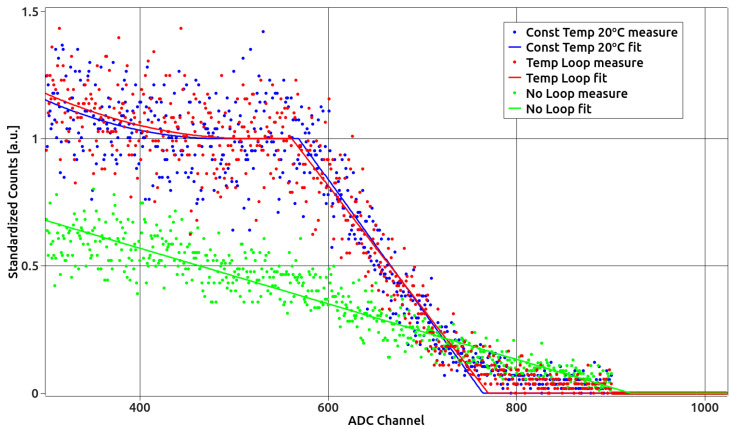
Comparison of the three measurements: with the TL disabled during temperature changes (green); with the TL enabled at a constant temperature of 20 °C (red); and with the TL enabled during temperature changes (dark blue).

When we used both increasing and decreasing temperature in a single measurement, the impact of these changes was much more difficult or impossible to observe, as their effects were the opposite. The temperature change was uniform and extended throughout the three-day measurement period. Figure 18 shows the effect of the threshold operation of the TL (step-wise voltage changes) much more clearly. To assess the effectiveness of the TL in our cosmic ray detector, we compare the Compton edge value. To facilitate a comparison, loop performance measurements were taken at constant temperatures of 20 and 25 °C (during this measurement, the loop did not change the supply voltage). Figure 20 shows a comparison of this measurement at a constant temperature of 20 °C as well as two other measurements where the temperature varied according to the cycle shown in Figure 18, with the TL enabled and disabled. The graph shows the qualitative difference between the measurement with the TL disabled and the measurements with the loop enabled. Measurements with the loop were enabled at a constant temperature of 20 °C, with the temperature varying continuously according to a preset program. We can clearly see that the absence of the TL or its malfunction will be clearly visible in the spectrum shape near the Compton edge. Determining the Compton edge value in such a situation is meaningless, and the resulting values will significantly differ from those in a properly functioning detector. From this figure, the measurements no longer show separate curves for the SiPM Master and Slave sensors (two ends of the detector board), only one example curve.

**Figure 21:**
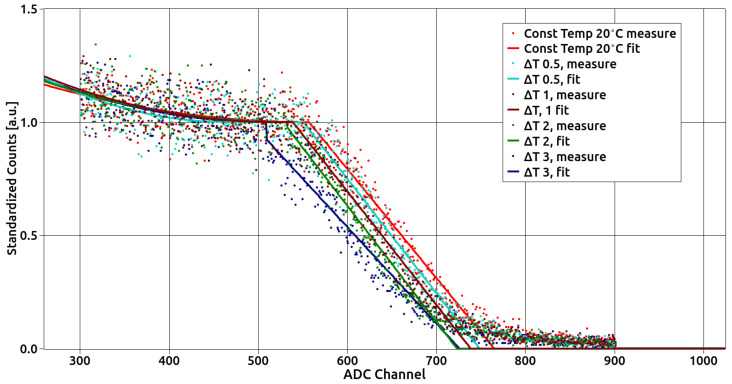
Comparison of the normalized spectrum graphs for measurements with the operating TL at different values of the threshold temperature (Dead Band), with the reference graph for the measurement with a constant temperature of 20 °C. When the dead-band increases, the measured value of the Compton edge shifts towards lower values. Averaging was performed using a weighted exponential average with a coefficient of $\alpha =10^{-5}$ and an averaging time of 0.1 s.

**Figure 22:**
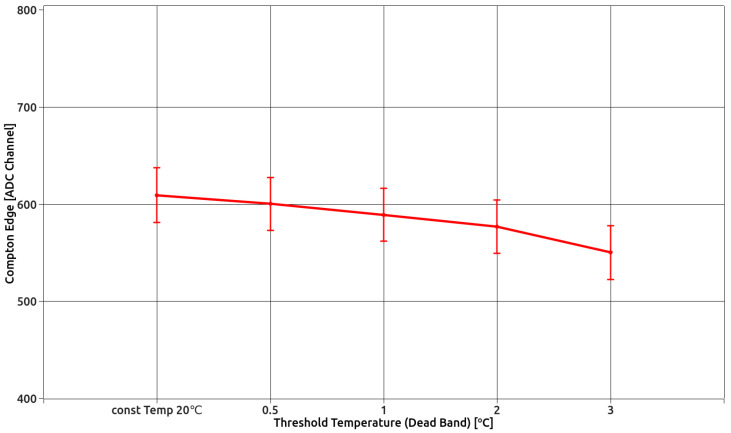
Dependence of the Compton edge value on the threshold temperature (Dead Band) parameter. When the dead-band increases, the measured value of the Compton edge shifts towards lower values.

Figure 21 shows a series of measurements taken at different threshold temperature values (dead band). The threshold value was varied from 0.5 °C to 3 °C. For a better comparison, the figure also includes a graph of the measurement at a constant temperature of 20 °C. We can clearly see that increasing this threshold value gradually increases the difference in the Compton Edge value (Figure 22). However, while this deviation is still relatively small for a threshold of 0.5 °C, it grows rapidly for larger values, exceeding 10% at 3 °C. We conclude that introducing such a threshold into the TL program is recommended and useful to prevent the loop from making small voltage corrections continuously; however, this threshold value should not be too high. At a threshold value of 0.5 °C, the difference in the Compton Edge value is less than 5%. Therefore, we recommend that this parameter be set to a value equal to or less than 0.5 °C.

**Figure 23:**
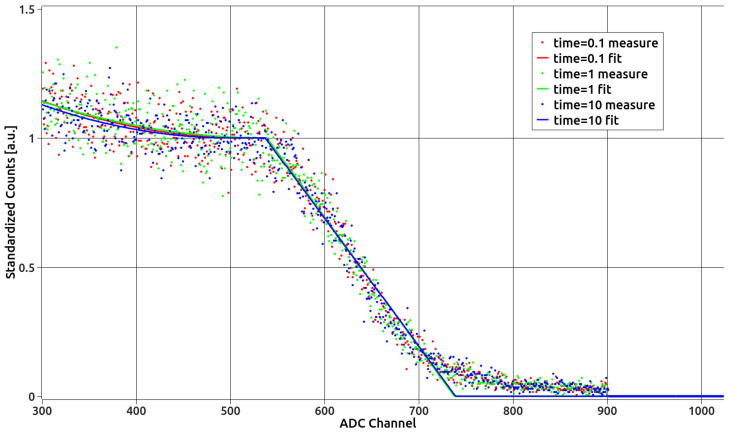
Comparison of the TL performance depending on different data collection times to calculate the average temperature (averaging times of 0.1 s, 1 s, and 10 s). The results within the measurement uncertainty limits coincide with each other. Averaging was performed using a weighted exponential average with a coefficient of $\alpha =10^{-5}$ and a temperature threshold step of 1 °C.

The next Figure 23 shows a series of measurements taken at different times during which the measured temperature values were collected and used to determine the average. The time values were 0.1, 1, and 10 s. We can clearly see that changing this time value does not noticeably affect the determined Compton Edge value and is not essential for the proper operation of our TL under the tested conditions. This parameter may be important for very rapid temperature changes, which are not normally encountered in laboratory or outdoor measurements.

**Figure 24:**
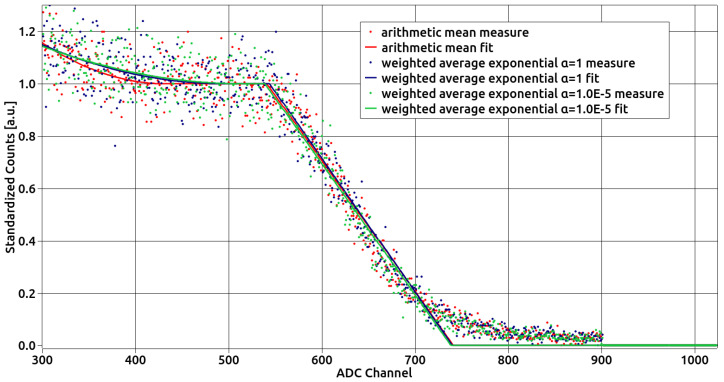
Comparison of the performance of the TL using three different methods for calculating the average temperature value: arithmetic mean, the exponentially weighted mean with an α coefficient of 1 (WE alpha 1), and the weighted mean with an α coefficient of $10^{-5}$. The results within the measurement uncertainty limits coincide with each other. Averaging was performed at a temperature threshold step of 1 °C and an averaging time of 0.1 s.

The last series of measurements compared various methods for determining the average temperature value (Figure 24). Many different average types were implemented as selectable options in the loop software (see Section 7). However, based on the simulations performed (Figure 17), it can be concluded that using some of them yields very similar values. For this reason, comparative measurements were performed for only three of them, which yielded significantly different results in the simulations. We compared the performance of the simplest method (arithmetic mean) with two types of exponential weighted average with alpha coefficients of 1 and $10^{-5}$. From the obtained measurement results, it is clear that changing the average calculation method also does not noticeably affect the determined Compton Edge value and is not essential for the proper operation of our TL under the tested conditions.

## 9. Summary

This article comprehensively presents all the issues related to the development of a properly and effectively operating TL in detectors using semiconductor photomultipliers (SiPM) for light reading. The use of a loop is essential to maintaining a constant detector signal amplification level under changing ambient temperatures. The design of the measurement setup, using a climatic chamber and small models of the actual detector, is described in detail, enabling reliable measurements. A fundamental parameter necessary for the operation of this type of detector is knowledge of the SiPM Temperature Coefficient Factor. We demonstrate a method for experimentally verifying this value provided by the SiPM sensor manufacturer and demonstrate that it is also necessary to take into account the influence of the electronics and the scintillator itself on the resulting value of this factor. In the case of our detector, this value changed by approximately 16%, from 50 mV/°C to 58 mV/°C, and this was was used in all subsequent measurements. To enable an effective comparison of the results of the TL, we decided to use the Compton edge value in the studied spectrum. A detailed description of how to use and determine the Compton edge value in the studied spectra is provided. The method we propose is based on fitting (using Mathematica [15] procedures) the function (Equation (1)) to the measurement data and, based on the coefficients obtained from this fit, calculating the Compton edge value (Equation (2)). Importantly, the developed method works effectively even with low measurement statistics. An analysis of potential measurement errors related to the method used was also conducted. Before the measurements began, a correction was made to the AFE electronics, significantly reducing the noise level and, consequently, the measurement uncertainty at extremely low currents. The AFE/HUB electronics control software was rebuilt and improved the functionality of the TL, allowing for the modification of several key parameters of this loop’s operation. The software was written using the Micropython environment for HUB and C utilizing the STM32F0 HAL for AFE. This article describes its basic features and the most important implemented variables. Thanks to all this work, a series of laboratory measurements were successfully conducted, demonstrating the effective operation of the designed TL during multi-day measurements and the impact of changes in key parameters. These measurements demonstrated that changing the temperature sample collection time for calculating the average value as well as the method of calculating the average value are of no significant importance. Therefore, the user can change these parameters without compromising the effective and correct operation of the TL. A crucial parameter is the temperature change threshold, after which the TL automatically changes the SiPM supply voltage to maintain constant system gain. It was shown that introducing such a threshold is essential to ensure that the system does not change the supply voltage continuously and frequently. However, this threshold should not exceed 0.5 °C to prevent the loop from negatively impacting the measurement system. This article is a supplement to the previously published article [7], which described the operation of the detector electronics and their calibration for correct measurement performance. Therefore, both articles can be considered as a set.

## Data Availability

Data can be made available upon request.

## Abbreviations

ADC	Analog Digital Converter
AFE	Analog Front End
CAN	Controller Area Network
CDR	Conceptual Design Report
DAC	Digital Analog Converter
DMA	Direct Memory Access
ED	Equivalent Detector
IWDG	Independent WatchDoG
LDO	Low DropOut regulator
MCORD	Modular Cosmic Ray Detector
MPD	Multi-Purpose Detector
NCBJ	National Centre for Nuclear Research
NICA	Nuclotron-based Ion Collider fAcility
REPL	Read, Eval, Print, Loop
SiPM	Silicon PhotoMultiplier
TDR	Technical Design Report
TL	Temperature Loop
